# Involvement of Kif4a in Spindle Formation and Chromosome Segregation in Mouse Oocytes

**DOI:** 10.14336/AD.2017.0901

**Published:** 2018-08-01

**Authors:** Feng Tang, Meng-Hao Pan, Yujie Lu, Xiang Wan, Yu Zhang, Shao-Chen Sun

**Affiliations:** College of Animal Science and Technology, Nanjing Agricultural University, Nanjing 210095, China.; College of Animal Science and Technology, Nanjing Agricultural University, Nanjing 210095, China.; College of Animal Science and Technology, Nanjing Agricultural University, Nanjing 210095, China.; College of Animal Science and Technology, Nanjing Agricultural University, Nanjing 210095, China.; College of Animal Science and Technology, Nanjing Agricultural University, Nanjing 210095, China.; College of Animal Science and Technology, Nanjing Agricultural University, Nanjing 210095, China.

**Keywords:** Kif4a, oocyte, meiosis, aneuploidy, aging

## Abstract

Kif4a, a member of the kinesin superfamily, has been reported to participate in a series of cellular processes such as chromosome condensation and cytokinesis during mitosis. However, the roles of KIF4a in meiosis are still unknown. In present study we found that the Kif4a protein expression decreased in maternal aged mouse oocytes. We then explored the roles of Kif4a in mouse oocyte meiosis by knockdown analysis. Kif4a was enriched at the spindle during mouse oocyte maturation. By specific knock down of the Kif4a using morpholino microinjection, we found that the disruption of Kif4a caused the failure of polar body extrusion. Further analysis indicated that Kif4a might affect the spindle morphology and chromosome alignment in the mouse oocytes, and this might be due to the regulation of tubulin acetylation. Moreover, our results showed that an increased proportion of aneuploidy in the Kif4a knock down oocytes, and this might be due to the loss of kinetochore-microtubule attachment. Taken together, these results suggested that Kif4a possibly regulated mouse oocyte meiosis through its effects on the spindle organization and accurate chromosome segregation, and the loss of Kif4a might be related with aneuploidy of aging oocytes.

In mammals, oocytes are characterized by unique asymmetric division to mediate their maturation [1]. During meiosis, the oocyte has the ability to produce a haploid gamete and is arrested in the middle of the second meiotic division (meiosis II) with fertilization competence [2]. During this process, genetic material is duplicated once and the oocyte becomes fully grown by undergoing two cell divisions, termed meiosis. The key events of meiosis I are chromosome segregation and extrusion of the first polar body [3]. Meiosis II is similar to somatic cell mitosis, and sister chromatids in the parental cell are equally divided between the two daughter cells. Moreover, the spindle is important for the equal distribution of genetic material. Spindle structure and chromosome segregation defects can result in aneuploidy [4, 5]. Fertilization of such aneuploid eggs may lead to embryonic aneuploidy, which is the main cause of miscarriage, implantation failure, and birth defects [6]. Meanwhile, such spindle and chromosome defects become more prevalent as maternal age increases and are considered the major factors resulting in birth defects and an increased incidence of miscarriage [7, 8]. Although previous studies showed that aging causes spindle defects and aneuploidy, the molecules/factors that participate in the aging-related process and its molecular mechanism are still largely unknown. In the present study, we found that Kif4a might be a critical factor for aging-related aneuploidy in mouse oocytes.

Kif4a is a member of the kinesin superfamily and belongs to the Kif4 subfamily, which also consists of three other members: Kif4b, Kif21a, and Kif21b [9]. In mammals, three Kif4 homologues (KLP3a, Xklp1, and human Kif4a [hKif4a]) have been identified in diverse species. Substantial studies have confirmed that the kinesin superfamily is implicated in regulating a range of cellular processes. For example, in Drosophila, KLP3a contributes to the establishment or stabilization of the central spindle [9], drives mitotic spindle pole separation during prometaphase and anaphase, and facilitates chromatid motility [10]. Moreover, Xklp1, the Kif4a homologue in *Xenopus*, is a major component required for mitotic chromosome assembly and is essential for chromosome positioning and spindle organization [11-13]. In addition, a recent study indicated that hKif4a is dominantly distributed in the nuclear matrix, is associated with chromosomes, and is a novel factor functioning in chromosome condensation and segregation during mitosis [14]. On the other hand, Kif4a affects chromosomes through its interaction with condensin complexes [15]. Like the abovementioned homologues, Kif4a affects chromosome congression and cytokinesis and can also control microtubule dynamics during anaphase and telophase, which are involved in proper spindle midzone formation [16-18]. During earlier stages of mitosis, Kif4a also immediately suppresses microtubule growth [19].

Although Kif4a has been implicated in multiple critical biological processes during mitosis, its physiological functions during oocyte meiosis have not been investigated. In this study, we found that Kif4a expression was decreased in aging mouse oocytes. Functional analysis employing morpholino knockdown (KD) showed that Kif4a regulated spindle organization and chromosome alignment/segregation in mouse oocytes, indicating that loss of Kif4a is one reason for aneuploidy in aging oocytes.

## MATERIALS AND METHODS

### Antibodies and chemicals

Rabbit polyclonal anti-Kif4a, mouse monoclonal anti-acetylated tubulin antibodies were from Santa Cruz (Santa Cruz, CA, USA). Human anti-centromere CREST antibody was purchased from Antibodies Incorporated (Davis, CA, USA). Mouse monoclonal anti-α-tubulin-FITC antibodies were from Sigma-Aldrich Corp. (St. Louis, MO, USA). FITC-conjugated and TRITC-conjugated goat anti-rabbit IgG, TRITC-conjugated goat anti-mouse IgG and TRITC- conjugated donkey anti-human IgG were from Zhongshan Golden Bridge Biotechnology, Co., Ltd. (Beijing). All other chemicals and reagents were from Sigma-Aldrich Corp., unless otherwise stated.

### Oocyte harvest and culture

ICR mice were used for all experiments. Our experiments were approved by the Animal Care and Use Committee of Nanjing Agriculture University and were performed in accordance with Animal Research Institute Committee guidelines. Mice were housed in a temperature-controlled room with an appropriate light: dark cycle, fed a regular diet, and maintained under the care of the Laboratory Animal Unit, Nanjing Agricultural University. Female mice (young 6-8weeks; old 42-45 weeks) were used for oocyte collection. To collect fully grown GV (germinal vesicle) oocytes, mice were superovulated with 5 IU pregnant mare serum gonadotropin (PMSG) by intraperitoneal injection, and 48 h later, cumulus-enclosed oocytes were obtained by manual rupturing of antral ovarian follicles. Cumulus cells were removed by repeatedly pipetting. For *in vitro* maturation, GV oocytes were cultured in M16 (Sigma) medium under mineral oil at 37°C in a 5% CO_2_ atmosphere.

### Nocodazole and Taxol treatment of oocytes

For nocodazole treatment, 10 mg/ml nocodazole in DMSO stock was diluted in M16 medium (Sigma) to give a final concentration of 20 μg/ml. After incubating in M16medium supplemented with nocodazole for 10min, the oocytes were collected for immunofluorescence microscopy after 9 h culture.

For taxol treatment, oocytes at the MI (metaphase I) stage were incubated in M16 medium containing 10μM taxol for 45 min.

### Kif4a antibody and Kif4a morpholino injection

Kif4a morpholino (MO) microinjection was used to knock down Kif4a in mouse oocytes. Kif4a-MO 5′-ATC CCC TTC ACC TCT TCT TTC ATG G-3′ (Gene Tools, Philomath, OR, USA) that targeted translation initiation was diluted with water to give a 300-nM stock solution, and 5-10 pl of MO solution was injected into oocytes. An MO standard control (5-10 pl) was injected as a control. For antibody injection, 5-10 pl of a Kif4a antibody was microinjected and the same volume of water was injected as a control.

After injection, the oocytes were cultured in M16 medium containing 5 μM milrinone for 24 h, and then washed three times, each for 2 min, in fresh M2 medium. The oocytes were then transferred to fresh M16 medium and cultured for 12 h to determine their maturation status (Pb1 extrusion) at 37 °C in a 5% CO2 atmosphere. Spindle and chromosome morphology were examined.

### Chromosome spread

Oocytes were exposed to 0.5% sodium citrate medium for 15mins. Then, oocytes were fixed in drop of methanol and glacial acetic acid (dilution ratio: methanol 3: glacial acetic acid 1) mixed medium on a glass slide. After air drying, chromosomes were stained with DAPI, and samples were examined under a laser scanning confocal microscope (Zeiss LSM 700 META, Germany).

### Cold Treatment

Metaphase I oocytes, which were briefly chilled at 4°C, immunstained with Crest to detect kinetochores, with tubulin antibody to visualize the spindles and counterstained with DAPI for chromosomes.

### Confocal microscopy

For single staining of Kif4a, ac-tubulin, α-tubulin, Crest staining, oocytes were fixed in 4% paraformaldehyde (in PBS) at room temperature for 30min and then permeabilized with 0.5% Triton X-100 in PBS for 20min. To reduce non-specific IgG binding, oocytes were blocked in blocking buffer (1% BSA-supplemented PBS) at room temperature for 1 h. For Kif4a or ac-tubulin staining, oocytes were incubated with a rabbit polyclonal anti-Kif4a antibody (1:50) or a mouse monoclonal anti- ac-tubulin antibody (1:100) at 4 °C overnight or at room temperature for 4 h. For Crest staining, oocytes were incubated with a Human anti-centromere CREST (1:200) at 4 ? for 48 h. After 3 washes (2 min each) with wash buffer (0.1% Tween 20 and 0.01% Triton X-100 in PBS), oocytes were labeled with an appropriate secondary antibody coupled to FITC-conjugated and TRITC-conjugated goat anti-rabbit IgG (1:100; for Kif4a staining), TRITC-conjugated goat-anti-mouse IgG (1:100; for ac-tubulin staining) or TRITC-donkey-anti-Human IgG at room temperature for 1 h. For α-tubulin-FITC staining, oocytes were incubated with an anti-α-tubulin-FITC antibody (1:100) at room temperature for 4 h. After 3 washes in wash buffer, oocytes were co-stained with DAPI to examine chromosomes. After staining, samples were mounted on glass slides and observed with a confocal laser-scanning microscope (Zeiss LSM 700 META, Germany). Furthermore, ImageJ software (National Institutes of Health, Bethesda, MD, USA) was used to quantify the intensity of fluorescence.

### Western blot analysis

A total of 180 mouse oocytes were placed in Laemmli sample buffer (SDS sample buffer with 2-mercaptoethanol) and heated at 100 ? for 10 mins. Proteins were separated by SDS-PAGE and then electrophoretically transferred to polyvinylidene fluoride (PVDF) membranes (Millipore, Billerica, MA, USA) at 20 V for 60 min. After transfer, membranes were blocked with PBST (PBS containing 0.1% Tween 20) that contained 5% non-fat milk for 1 h, followed by incubation at 4 °C overnight with a rabbit polyclonal anti-Kif4a (1:200), a rabbit monoclonal anti-αtubulin antibody (1:2000) and a mouse monoclonal anti-ac Tubulin antibody (1:1000). After washing 3 times in PBST (10 min each), membranes were incubated at 37 °C for 1 h with HRP-conjugated Pierce Goat anti-Rabbit IgG (1:6000) or HRP-conjugated Pierce Goat anti-mouse IgG (1:1000). Finally, the specific proteins were visualized using chemiluminescence reagent (Millipore, Billerica, MA).

### Statistical analysis

At least three biological replicates were used for each analysis. Each replicate was done by an independent experiment at the different time. Results are given as means ± SEM. Statistical comparisons were made using analysis of variance (ANOVA) and differences between treatments groups were assessed with Duncan’s multiple comparisons test. A p-value of < 0.05 was considered significant.

## RESULTS

### Kif4a expression decreases with maternal aging in mouse oocytes

**Figure 1.**
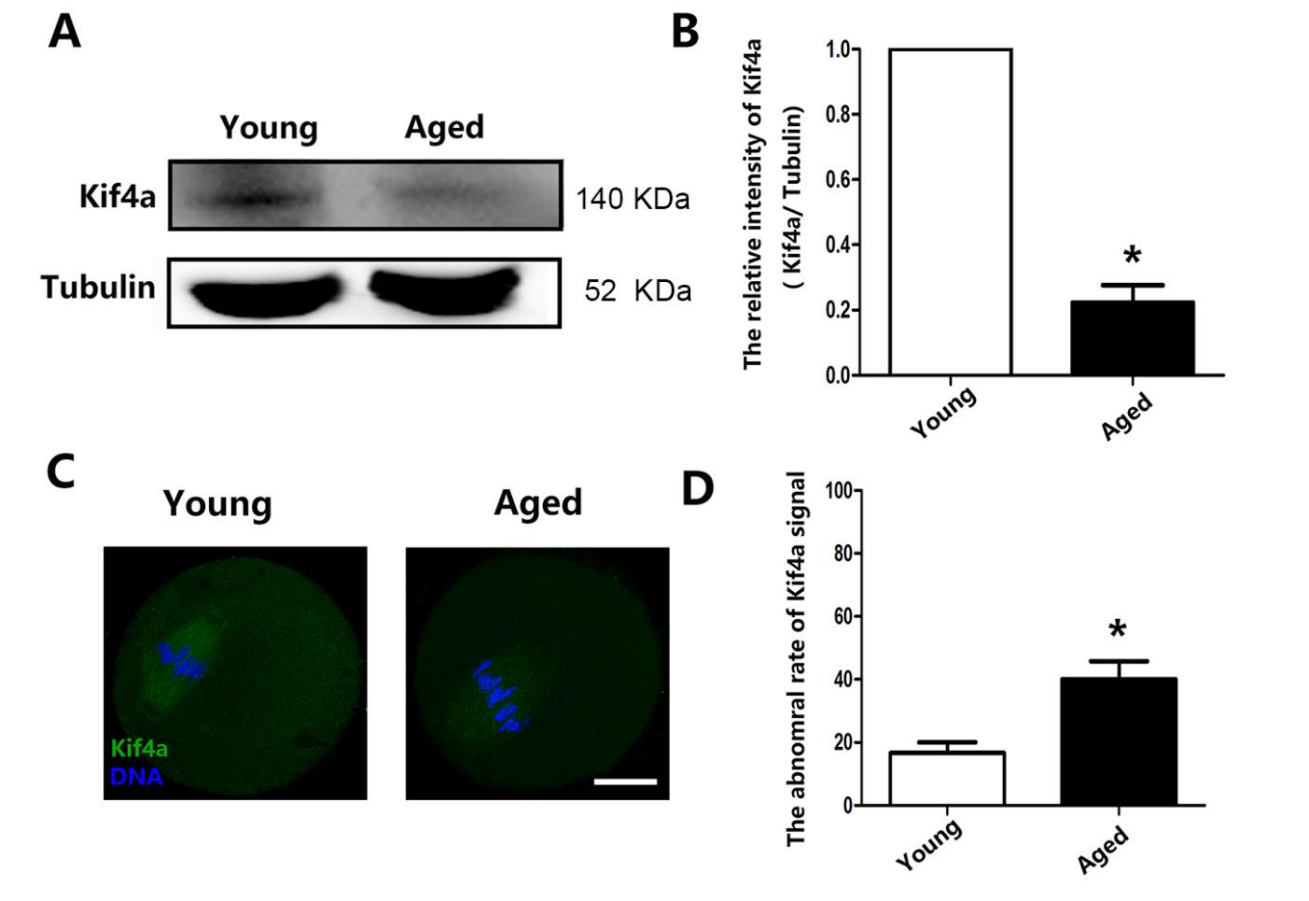
Kif4a expression decreases in mouse oocytes with maternal aging (**A**) Kif4a expression examined by western blotting. Three independent experiments were performed. (**B**) Relative intensities of the Kif4a protein bands in young and aged oocytes. Kif4a expression was significantly decreased in aged oocytes (Kif4a:tubulin, 1.0 vs. 0.223 ± 0.053; *p < 0.05). (**C**) Representative images of young and aged oocytes stained with an anti-Kif4a antibody. Green, Kif4a; blue, DNA. Scale bars, 20 μm. (**D**) Percentages of young and aged oocytes with abnormal Kif4a signals. Data are presented as mean percentage (± SEM) of at least three independent experiments. The asterisk denotes *p* < 0.05.

We first examined Kif4a protein expression in aging mice by western blot analysis. Kif4a protein expression was lower in oocytes of aging mice than in those of young mice (Fig. 1A). This was confirmed by calculation of the relative densities of the protein bands (1.0 vs. 0.223 ± 0.053; *p* < 0.05; Fig. 1B). We analyzed Kif4a expression by immunofluorescence staining. In the oocytes of young mice, the Kif4a was localized at the spindle, while compared with oocytes of young mice; there was barely any signal in oocytes of aging mice (Fig. 1C). The percentage of oocytes with abnormal Kif4a signals was significantly higher among aging oocytes than among young oocytes (16.67% ± 3.33%, n=30 vs. 40.00% ± 5.77%, n=30, *p* < 0.05) (Fig. 1D). These results revealed that Kif4a expression was reduced in aging oocytes.

### Kif4a is enriched at the meiotic spindle in mouse oocytes

**Figure 2.**
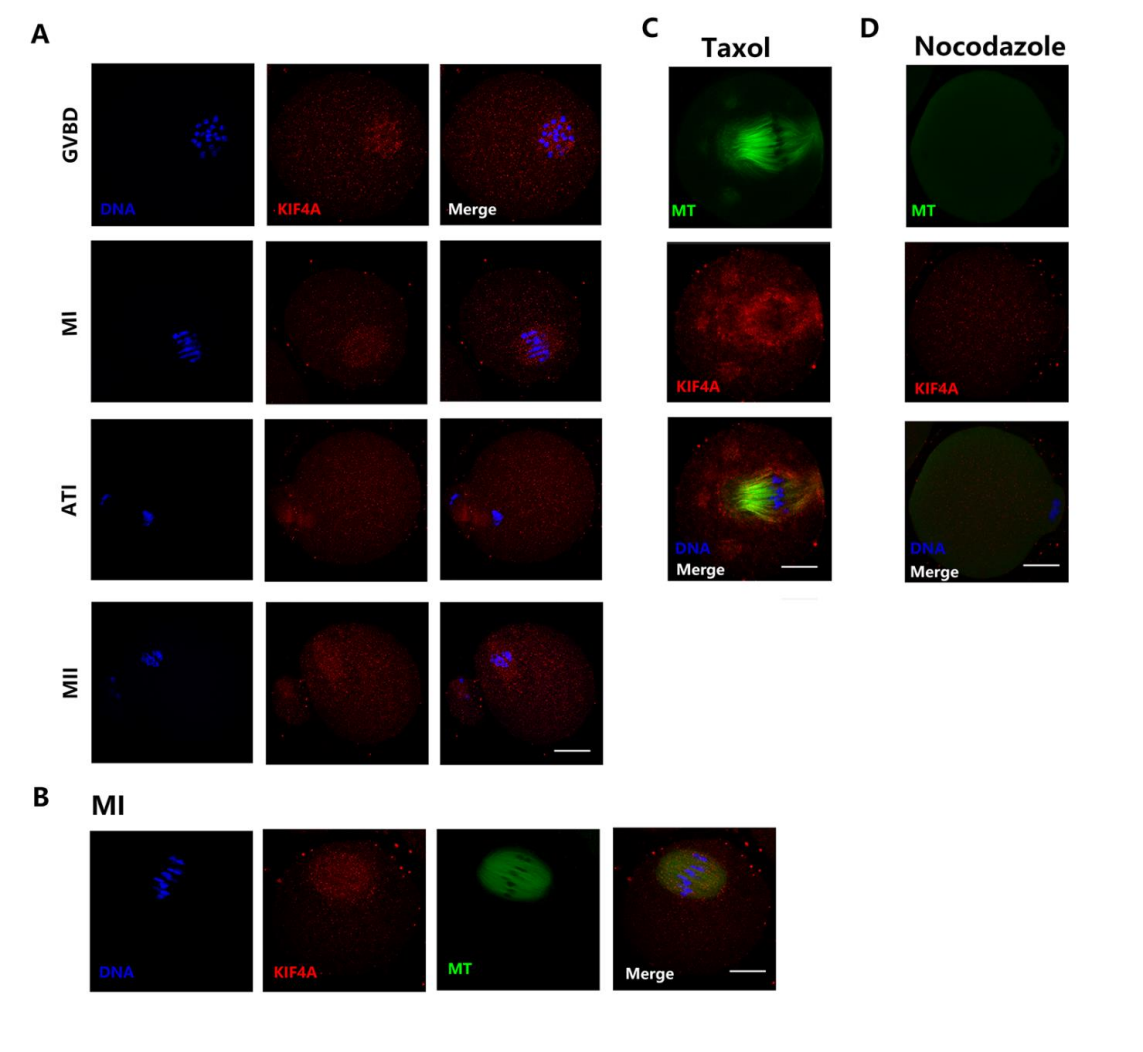
Localization of Kif4a in mouse oocytes (**A**) Oocytes at different stages were immunolabeled with an anti-Kif4a antibody (red) and counterstained with DAPI to visualize DNA (blue). Kif4a was primarily accumulated around chromosomes after GVBD. Kif4a was accumulated at the meiotic spindle region at both the MI and MII stages. Kif4a localized at the midbody at the ATI stage. (**B**) Double staining of MI oocytes with an anti-Kif4a antibody (red) and an anti-α-tubulin antibody (green). Oocytes were counterstained with DAPI to visualize DNA (blue). Kif4a mainly localized on the meiotic spindle. (**C**) Subcellular localization of Kif4a after taxol treatment during mouse oocyte meiotic maturation. Green, α-tubulin; red, Kif4a. (**D**) Subcellular localization of Kif4a after nocodazole treatment during mouse oocyte meiotic maturation. Green, α-tubulin; red, Kif4a. Scale bars, 20 μm.

Next, the subcellular localization of Kif4a at different stages of meiotic maturation was examined by performing immunofluorescence staining with an anti-Kif4a antibody. Kif4a was primarily accumulated around chromosomes after germinal vesicle breakdown (GVBD) (Fig. 2A). At metaphase I (MI), Kif4a was accumulated at the meiotic spindle region. When oocytes progressed to anaphase-telophase I (ATI), Kif4a mainly assembled at the midbody. At metaphase II (MII), Kif4a was enriched at the spindle. Double staining of MI oocytes with an anti-Kif4a antibody and an anti-α-tubulin antibody demonstrated that the localization of Kif4a was similar to that of microtubules (Fig. 2B). We also examined the localization of Kif4a after taxol or nocodazole treatment. Upon taxol treatment, microtubule polymerization was promoted and Kif4a was enriched around the spindle (Fig. 2C). However, Kif4a dispersed into the cytoplasm and the spindle disassembled upon nocodazole treatment (Fig. 2D).

### Kif4a KD affects mouse oocyte maturation

**Figure 3.**
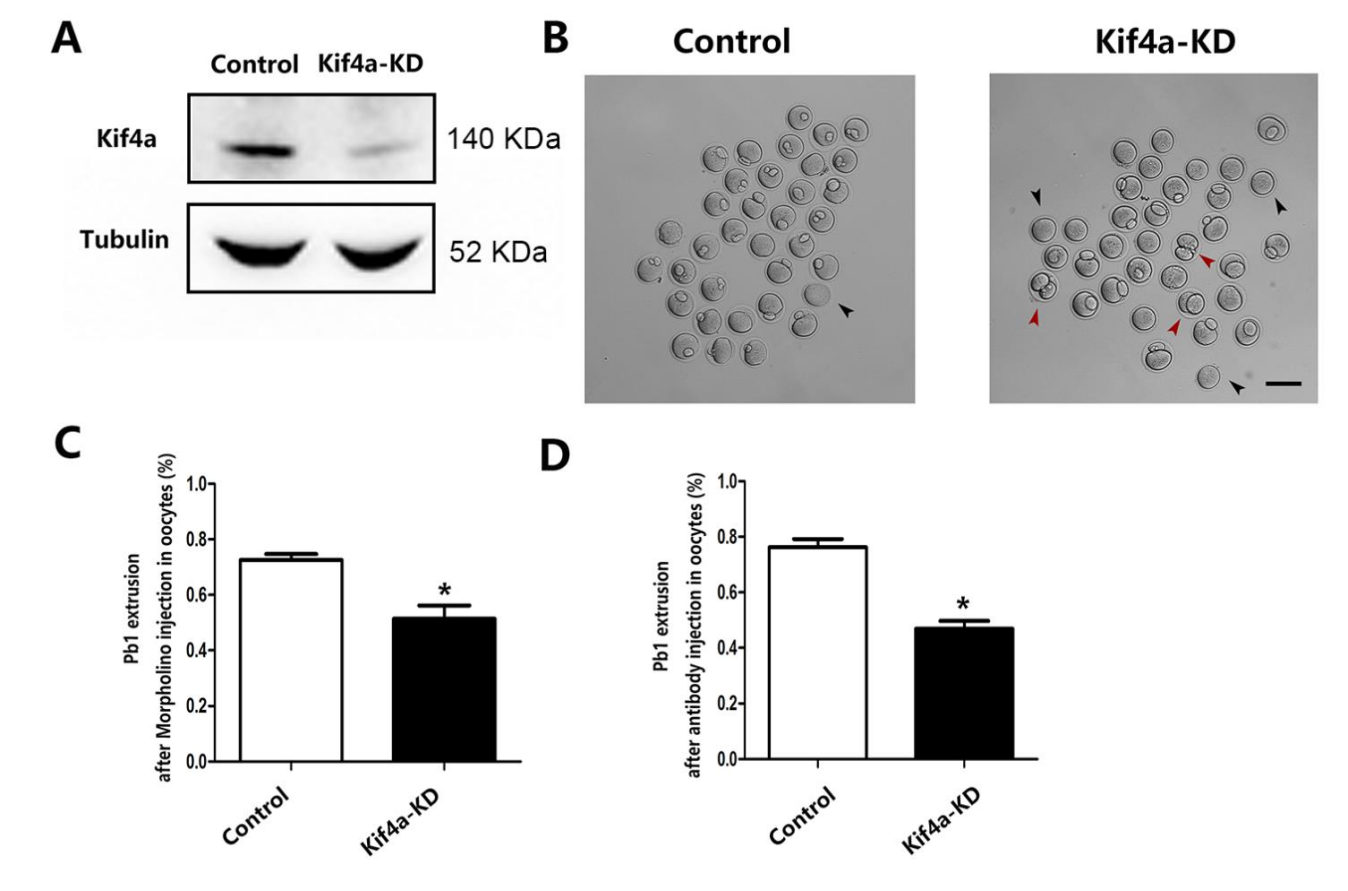
Effects of Kif4a KD on oocyte maturation (**A**) KD of endogenous Kif4a protein expression after Kif4a morpholino injection was verified by western blot analysis. Three independent experiments were performed. (**B**) Phase-contrast images of control morpholino-injected and Kif4a-KD oocytes after culture for 12 h. Black arrowheads indicate oocytes that failed to extrude a polar body. Red arrowheads indicate oocytes with an apparently large polar body. Scale bar, 100 μm. (**C**) The rate of pbI in control and Kif4a-KD oocytes. (**D**) The rate of pbI in control and antibody-injected oocytes. Data are presented as mean percentage (±SEM) of at least three independent experiments. The asterisk denotes *p* < 0.05.

To investigate the potential functions of Kif4a, we employed morpholino and antibody microinjection to disrupt Kif4a activity. Western blotting confirmed that Kif4a protein expression was significantly reduced following Kif4a morpholino injection (Fig. 3A). We observed abnormal polar body extrusion (pbI) upon Kif4a KD. A number of Kif4a-KD oocytes failed to extrude the polar body or had large polar bodies (Fig. 3B). The pbI rate was lower in Kif4a-KD oocytes than in control oocytes (49.8% ± 5.80%, n=228 vs. 72.6% ± 2.14%, n=261, control, *p* < 0.05; Fig. 3C). We acquired similar results following antibody injection to disrupt Kif4a activity; the pbI rate was significantly lower in Kif4a-disrupted oocytes than in control oocytes (51.2% ± 3.76%, n=188 vs. 76.1% ± 3.04%, n=240, control, *p* < 0.05; Fig. 3D).

### Kif4a KD affects spindle formation and chromosome alignment in mouse oocytes

**Figure 4.**
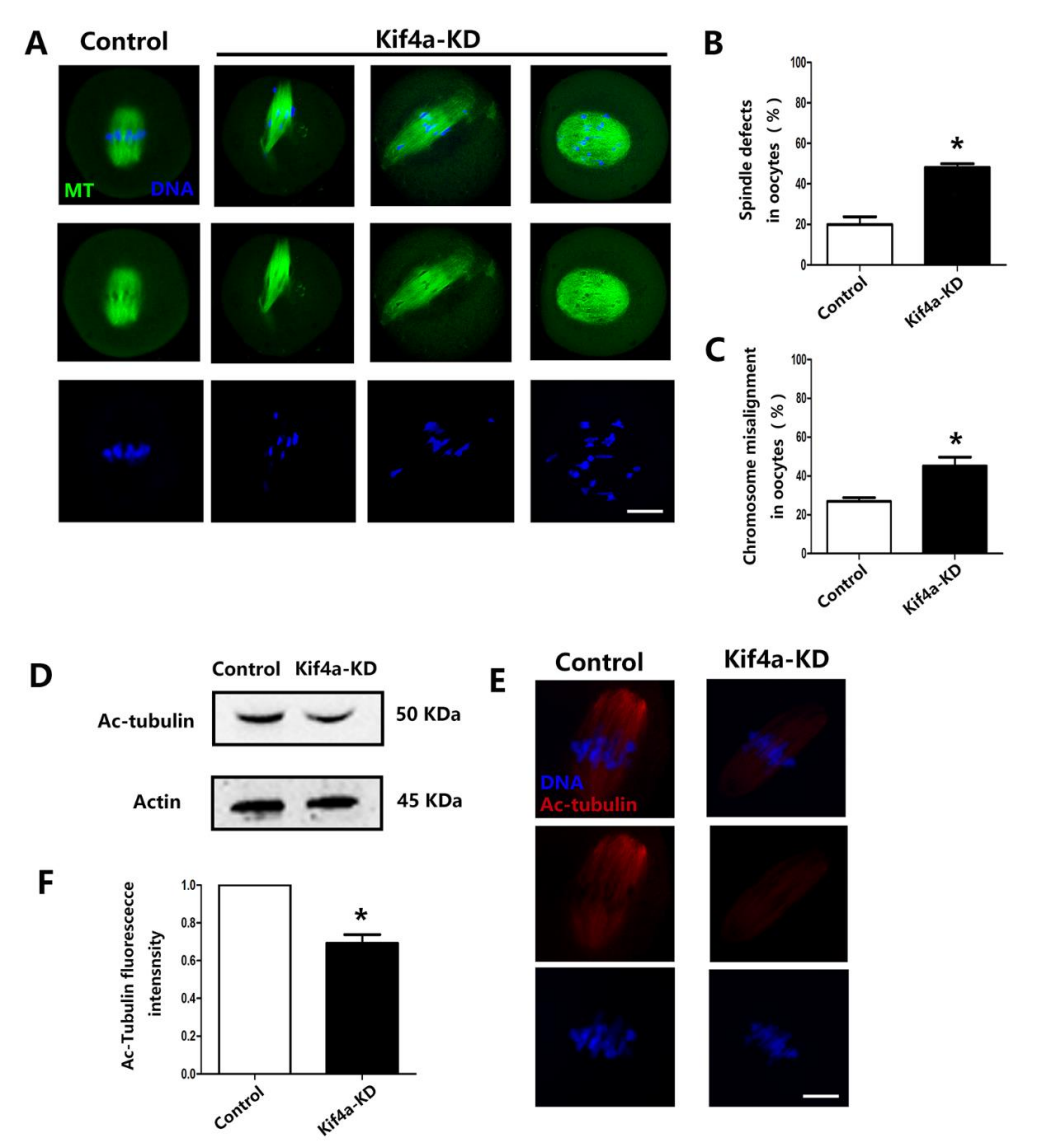
Kif4a KD causes spindle defects and chromosome misalignment during oocyte meiosis (**A**) Control morpholino-injected and Kif4a-KD oocytes were stained with an anti-α-tubulin antibody to visualize the spindle (green) and counterstained with DAPI to visualize chromosomes (blue). Control MI oocytes had a typical barrel-shape spindle and well-aligned chromosomes at the metaphase plate. Meanwhile, Kif4a-KD oocytes had several spindle defects, such as malformed spindles, elongated spindles, and inflated spindles. Scale bars, 20 μm. Three independent experiments were performed. (**B**) Percentages of control and Kif4a-KD oocytes with aberrant spindles. (**C**) Percentages of control and Kif4a-KD oocytes with misaligned chromosomes. Data are presented as mean percentage (±SEM) of at least three independent experiments. The asterisk denotes *p* < 0.05. (**D**) Endogenous ac-tubulin protein expression after Kif4a morpholino injection was verified by western blot analysis. Three independent experiments were performed. (**E**) Control and Kif4a-KD oocytes were stained with an anti-ac-α-tubulin antibody (red) to visualize ac-microtubules and with DAPI to visualize chromosomes. The ac-α-tubulin signal was significantly decreased in Kif4a-KD oocytes. Three independent experiments were performed. (**F**) Quantification of the data showed that the ac-tubulin fluorescence signal intensity was decreased in Kif4a-KD oocytes. At least 30 oocytes were analyzed for each group. The asterisk denotes *p* < 0.05.

Based on its localization pattern, we hypothesized that Kif4a might have a regulatory function in spindle-related cellular processes. Thus, we examined spindle morphology in Kif4a-KD oocytes. Most control oocytes had typical barrel-shaped spindles and well-aligned chromosomes at the metaphase plate (Fig. 4A). By contrast, Kif4a-KD oocytes exhibited several abnormal spindle phenotypes, such as malformed spindles, elongated spindles, and inflated spindles (Fig. 4A). Moreover, chromosomes were severely misaligned. The incidence of spindle defects was significantly higher in Kif4a-KD oocytes than in control oocytes (48.0% ± 1.94%, n=158 vs. 19.9% ± 3.98% control, n=95, *p* < 0.05; Fig. 4B). Similarly, the incidence of chromosome misalignment was higher in Kif4a-KD oocytes than in control oocytes (45.1% ± 4.56%, n=158 vs. 26.9% ± 1.84% control, n=95, *p* < 0.05; Fig. 4C). The level of tubulin acetylation is important for spindle formation and chromosome congression. To investigate the causes of spindle defects upon Kif4a KD, we assessed endogenous acetylated (ac) tubulin expression by western blotting. Ac-tubulin protein expression was lower in Kif4a-KD oocytes than in control oocytes (Fig. 4D). To further confirm this, we performed staining with an anti-Ac-tubulin antibody. Fluorescence signals were significantly weaker in Kif4a-KD oocytes than in control oocytes (Fig. 4E). Moreover, the fluorescence intensity of ac-tubulin was also reduced (1.0 vs. 0.692 ± 0.046, *p* < 0.05; Fig. 4F), which is consistent with the western blot data.

### Kif4a KD affects kinetochore-microtubule attachment and induces aneuploidy in mouse oocytes

**Figure 5.**
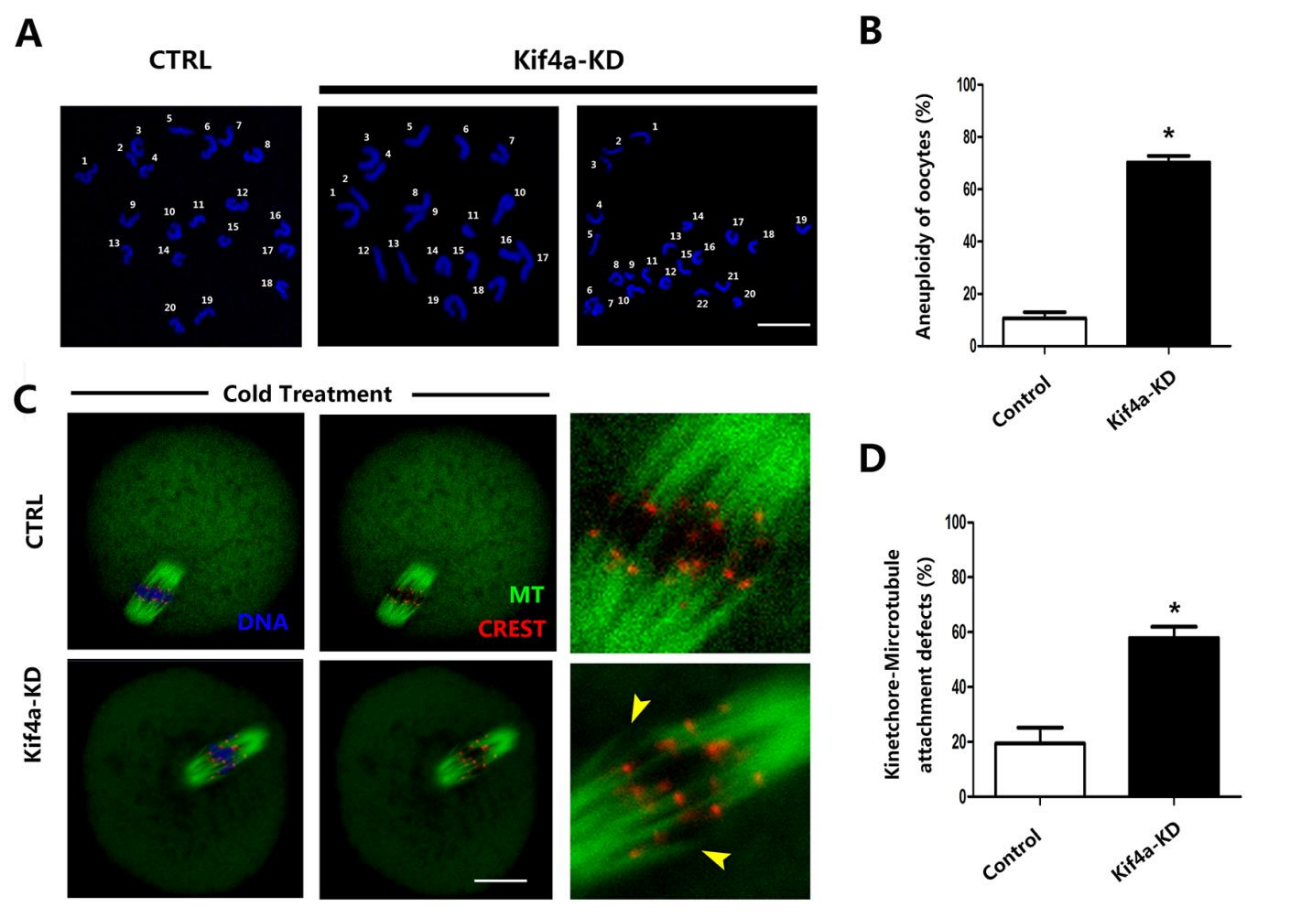
Kif4a KD induces aneuploidy and affects kinetochore-microtubule attachment in mouse oocytes (**A**) Chromosome spreads of control and Kif4a-KD MII oocytes. Representative images of euploid control oocytes and aneuploid Kif4a-KD oocytes are shown. (**B**) Percentages of aneuploid control and Kif4a-KD oocytes. Scale bars, 20 μm. *p* < 0.05. Data are presented as mean percentage (±SEM) of at least three independent experiments. (**C**) MI oocytes were subjected to cold treatment to depolymerize unstable microtubules that were not attached to kinetochores. Control and Kif4a-KD oocytes were stained with an anti-α-tubulin antibody to visualize the spindle (green) and with Crest to visualize kinetochores. Oocytes were counterstained with DAPI to visualize chromosomes (blue). Yellow arrowheads indicate the absence of kinetochores or the unattached kinetochore. (**D**) Percentages of control and Kif4a-KD oocytes with kinetochore-microtubule attachment defects. At least 30 oocytes were analyzed for each group. Data are presented as mean percentage (±SEM) of at least three independent experiments. The asterisk denotes *p* < 0.05.

The misalignment of chromosomes generally causes aneuploidy, an incorrect number of chromosomes in eggs, which might lead to miscarriage, embryonic lethality, or genetic disorders. To confirm this possibility, we performed karyotype analysis of MII oocytes by chromosome spreading. Kif4a KD led to the generation of aneuploid eggs, i.e., those with a gain or loss of chromosomes (Fig. 5A). The incidence of aneuploidy was significantly higher in Kif4a-KD oocytes than in control oocytes (70.30% ± 2.46%, n=37 vs. 10.69% ± 2.36% control, n=37, *p* < 0.01) (Fig. 5B).

To determine whether the misalignment of chromosomes observed after KD of Kif4a is caused by the defective interaction between kinetochores and microtubules, we assessed the stability of kinetochore-microtubule attachments by performing cold treatment to depolymerize unstable microtubules that were not attached to kinetochores. Kinetochores were clearly attached to microtubules in control oocytes, whereas kinetochores cannot match the corresponding microtubules in Kif4a-KD oocytes (Fig. 5C). The incidence of kinetochore-microtubule defects was higher in Kif4a-KD oocytes than in control oocytes (57.88% ± 4.08%, n=31 vs. 19.39% ± 5.81% control, n=31, *p* < 0.05; Fig. 5D).

## DISCUSSION

In the present study, we found that Kif4a expression was decreased in oocytes of aging mice. We then attempted to investigate Kif4a functions and their relationship with aneuploidy in female meiosis. Loss of Kif4a affected mouse oocyte maturation. Further investigations revealed increases in spindle defects and chromosome misalignment in Kif4a-KD oocytes, together with deficient kinetochore-microtubule attachment and an increased incidence of aneuploidy. Our results provide evidence that Kif4a is important for aging-related aneuploidy in mouse oocytes.

Recent research indicated that Kif4 associates with chromosomes throughout mitosis, but still appears at the spindle region during anaphase in Hela cells [17, 20]. Similar to the localization of Kif4, Kif4a also accumulates around the central spindle during anaphase in Hela cells [21]. In the present study, Kif4a mainly assembled around chromosomes after GVBD, while it was enriched at the meiotic spindle at the MI and MII stages. In addition, Kif4a was present at the midzone during the ATI stage. Our results showed that Kif4a was associated with microtubules throughout the entire oocyte maturation process, which suggests it is involved in a spindle-related process during mouse oocyte meiosis. We then explored Kif4a functions by performing KD analysis. pbI failed upon Kif4a KD, indicating that Kif4a is necessary for mouse oocyte maturation.

Microtubules are important cytoskeletal polydimers with vital functions, including modulation of cell shape, cell transport, cell motility, and cell division [22]. Spindle assembly and function depend on regulation of microtubule organization. Kinesin families regulate spindle formation in different models. Recent studies report that Kif25 affects maintenance of spindle stability during mitosis in Hela cells [23]. Furthermore, Kif14 can inhibit minus-end-directed microtubule motility [24], and Eg5 (also known as Kif11) affects spindle assembly and spindle bipolarity in Hela cells [25]. In *Xenopus* oocytes, Kif18a is indispensable for the correct function of meiotic spindles [26]. In *Caenorhabditis elegans* embryos, depletion of KLP-7^MCAK^ (a member of the kinesin-13 family) delays spindle and sister chromatid segregation and prevents assembly of astral microtubules [27]. Moreover, hKif4a regulates spindle structure in MRC-5 cells [28]. Our data showed that specific KD of Kif4a caused spindle defects and chromosome misalignment in oocytes, indicating that Kif4a has conserved roles in different species or models. We next tried to elucidate how Kif4a regulates spindles in oocytes. Post-translational modifications of tubulin are crucial for spindle formation. These modifications include detyrosination/tyrosination, acetylation/deacetylation, phosphorylation, polyglutamylation, and polyglycylation [29]. ac-α-tubulin is present in various microtubule structures, including primary cilia, centrioles, and the mitotic spindle, and may have a role in stabilizing these structures [30]. In addition, ac-tubulin has been found in oocytes [31, 32]. Our data showed that specific KD of Kif4a reduced acetylation of tubulin in oocytes. Therefore, we hypothesize that Kif4a might affect the level of tubulin acetylation, which could further disrupt spindle morphology in mouse oocytes.

Misaligned chromosomes might induce aneuploidy. To test this, we examined kinetochore-microtubule attachment in mouse oocytes. For correct chromosome segregation, bundles of microtubules (K-fibers) emanating from opposite spindle poles must attach to each of two sister kinetochores [33]. In oocytes, kinetochore-microtubule attachment also has a vital function. Recent research indicates that loss of Klp6 (a member of the kinesin-8 family) results in aberrant chromosome pushing movements in fission yeast [34]. Moreover, Kif15 assembles on kinetochore fibers in Hela cells, with the antagonism competence of centrosome separation [35]. Disruption of Kif2a or Kif1b activity can cause aberrant chromosome alignment in mouse oocytes [36, 37]. Our data demonstrated that Kif4a KD caused a high incidence of aneuploidy in mouse oocytes. Further analysis indicated that Kif4a KD affected kinetochore-microtubule attachment in oocytes. These results suggest that Kif4a affects kinetochore-microtubule attachment, which further regulates chromosome alignment and accurate segregation in mouse oocytes.

Our results indicated that Kif4a KD could induce aneuploid eggs with spindle defects and chromosome misalignment. In human oocytes, aneuploidy increases with maternal aging [38], and chromosome number abnormalities are the main causes of miscarriage, mental retardation, and congenital defects [39]. In mammals, an age-related decrease in fertility is mainly caused by reduced developmental competence, particularly with spindle and chromosome abnormalities [40]. Recent evidence showed that reproductive aging is mediated by a number of factors within the nucleus, including disruption of cohesions, a reduction in chiasma, aneuploidy, disruption of meiotic spindles, and DNA damage caused by chronic exposure to reactive oxygen species [41]. In the present study, Kif4a protein expression was lower in aged oocytes than in control oocytes. Based on the spindle and chromosome defects in Kif4a-KD oocytes, we propose that Kif4a is a key factor that regulates maternal age-associated meiotic defects.

In conclusion, our results indicate that Kif4a affects mouse oocyte maturation via its effects on spindle and chromosome organization, and loss of Kif4a might be an important cause of maternal age-associated meiotic defects.
